# PLA_2_-Triggered Activation of Cyclosporine-Phospholipid Prodrug as a Drug Targeting Approach in Inflammatory Bowel Disease Therapy

**DOI:** 10.3390/pharmaceutics14030675

**Published:** 2022-03-18

**Authors:** Milica Markovic, Shimon Ben-Shabat, Jagadeesh Nagendra Manda, Karina Abramov-Harpaz, Clil Regev, Yifat Miller, Aaron Aponick, Ellen M. Zimmermann, Arik Dahan

**Affiliations:** 1 Department of Clinical Pharmacology, School of Pharmacy, Faculty of Health Sciences, Ben-Gurion University of the Negev, Beer-Sheva 8410501, Israel; milica@post.bgu.ac.il (M.M.); sbs@bgu.ac.il (S.B.-S.); 2 Department of Chemistry, University of Florida, Gainesville, FL 32603, USA; jagadeesh.manda@chem.ufl.edu (J.N.M.); aaron.aponick@gmail.com (A.A.); 3 Department of Chemistry, Ben-Gurion University of the Negev, Beer-Sheva 8410501, Israel; karinaab@post.bgu.ac.il (K.A.-H.); regevcl@post.bgu.ac.il (C.R.); ymiller@bgu.ac.il (Y.M.); 4 Ilse Katz Institute for Nanoscale Science and Technology, Ben-Gurion University of the Negev, Beer-Sheva 8410501, Israel; 5 Department of Medicine, Division of Gastroenterology, University of Florida, Gainesville, FL 32610, USA; ellen.zimmermann@medicine.ufl.edu

**Keywords:** inflammatory bowel disease, drug targeting, oral drug delivery, prodrug, cyclosporine, phospholipase A_2_, colonic brush border membrane vesicles

## Abstract

Oral medication with activity specifically at the inflamed sites throughout the gastrointestinal tract and limited systemic exposure would be a major advance in our therapeutic approach to inflammatory bowel disease (IBD). For this purpose, we have designed a prodrug by linking active drug moiety to phospholipid (PL), the substrate of phospholipase A_2_ (PLA_2_). PLA_2_ expression and activity is significantly elevated in the inflamed intestinal tissues of IBD patients. Since PLA_2_ enzyme specifically hydrolyses the *sn*-2 bond within PLs, in our PL-based prodrug approach, the *sn*-2 positioned FA is replaced with cyclosporine, so that PLA_2_ may be exploited as the prodrug-activating enzyme, releasing the free drug from the PL-complex. Owing to the enzyme overexpression, this may effectively target free cyclosporine to the sites of inflammation. Four PL-cyclosporine prodrugs were synthesized, differing by their linker length between the PL and the drug moiety. To study the prodrug activation, a novel enzymatically enriched model was developed, the colonic brush border membrane vesicles (cBBMVs); in this model, tissue vesicles were produced from colitis-induced (vs. healthy) rat colons. PLA_2_ overexpression (3.4-fold) was demonstrated in diseased vs. healthy cBBMVs. Indeed, while healthy cBBMVs induced only marginal activation, substantial prodrug activation was evident by colitis-derived cBBMVs. Together with the PLA_2_ overexpression, these data validate our drug targeting strategy. In the diseased cBBMVs, quick and complete activation of the entire dose was obtained for the 12-carbon linker prodrug, while slow and marginal activation was obtained for the 6/8-carbon linkers. The potential to target the actual sites of inflammation and treat any localizations throughout the GIT, together with the extended therapeutic index, makes this orally delivered prodrug approach an exciting new therapeutic strategy for IBD treatment.

## 1. Introduction

Inflammatory bowel disease (IBD) encompasses Crohn’s disease and ulcerative colitis, two chronic, relapsing, inflammatory conditions of the gastrointestinal tract with increasing global incidence and high disease burden [1]. Over five million adults and children in the world suffer from IBD, and endure difficult symptoms such as abdominal pain, diarrhea, rectal bleeding, and many others [2,3]. IBD is difficult to manage, and comes with great costs of diagnostic procedures, drug therapy, side effects management, hospitalization, and loss of productivity. It is typically diagnosed in the second or third decades of life, it is life-long, and there is no cure. The main goal of current therapeutic strategies is to reduce the inflammation [4]. To date, drug delivery strategies in IBD, such as rectal drug product and oral dosage forms, target a general intestinal region (mainly the colon/lower ileum) [5,6,7]. Challenges to effective delivery in the hostile intestinal luminal environment are met by two general approaches: chemical modification of the drug and formulation-based technologies. Formulation approaches that are employed to achieve this goal include rectal products, or formulations that rely on pH-, time-, microflora, and pressure-dependent drug release [8,9]. In cases when inflammation is outside the particular targeted region (e.g., upper small intestine in Crohn’s disease patients), these delivery strategies will not be effective; even if the lesions are within the targeted intestinal region, this strategy essentially leads to a suboptimal pharmacokinetic and/or pharmacodynamic profile, as only a portion of the drug reaches the actual site(s) of inflammation, whereas the rest is delivered to healthy tissues and may cause serious side effects [10]. In this work, we present an example of the chemical modification approach, a novel prodrug strategy that may enable drug targeting directly to the inflamed patches; activity towards inflamed sites throughout the gastrointestinal tract (GIT) and limited systemic exposure would be a major advance in our therapeutic approach to IBD.

**Scheme 1:**
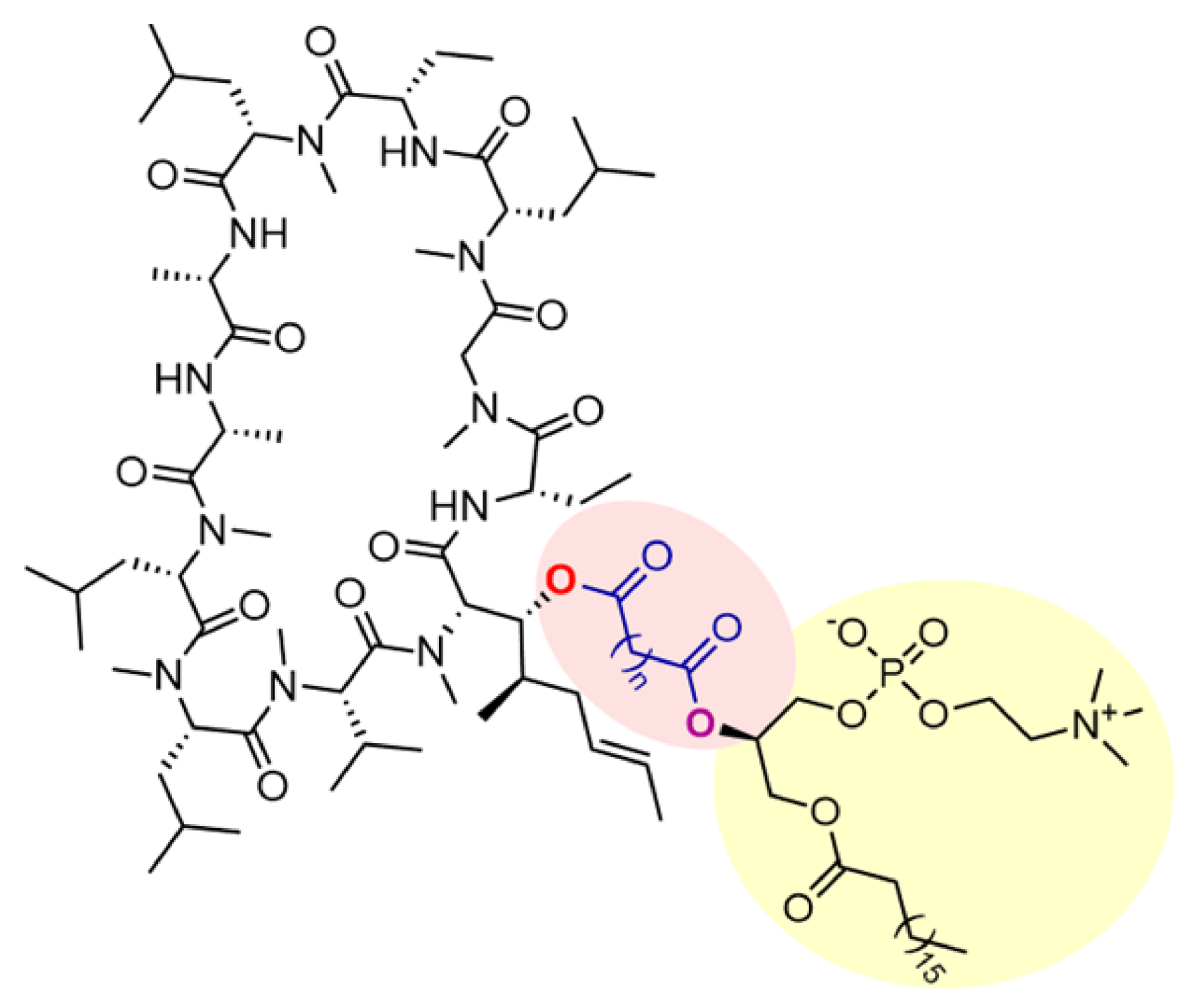
The structure of PL–cyclosporine prodrugs with increasing linker length (n = 6, 8, 10 and 12 -CH_2_ units) between the PL and cyclosporine.

Cyclosporine has well-studied anti-inflammatory activity. In patients with IBD, cyclosporine has been used to treat refractory or severely active disease by selectively inhibiting T-cell mediated cytokine production [11]. It binds to cyclophilin and acts to block the phosphatase activity of calcineurin which inhibits T-cell mediated cytokine production [12]. It is currently used topically and systemically to treat a variety of diseases such as psoriasis, uveitis, and graft vs. host disease [13,14,15]. The use of cyclosporine however, is limited to the most severe cases due to substantial systemic toxicities and narrow therapeutic window; systemic bioavailability, the usual goal of most orally delivered drugs, is responsible for the serious side effects of cyclosporine and other systemically available immunosuppressant drugs [16]. A few centers continue to use cyclosporine for short term use in patients who are hospitalized with severely active ulcerative colitis [17]. Improved, safer alternatives in drug delivery of immunosuppressant drugs are greatly needed to circumvent serious side effects, particularly for long-term maintenance therapy in patients treated for this devastating disease. For these reasons, we have selected cyclosporine as the drug entity in our prodrug design, Scheme 1 [18].

**Figure 1:**
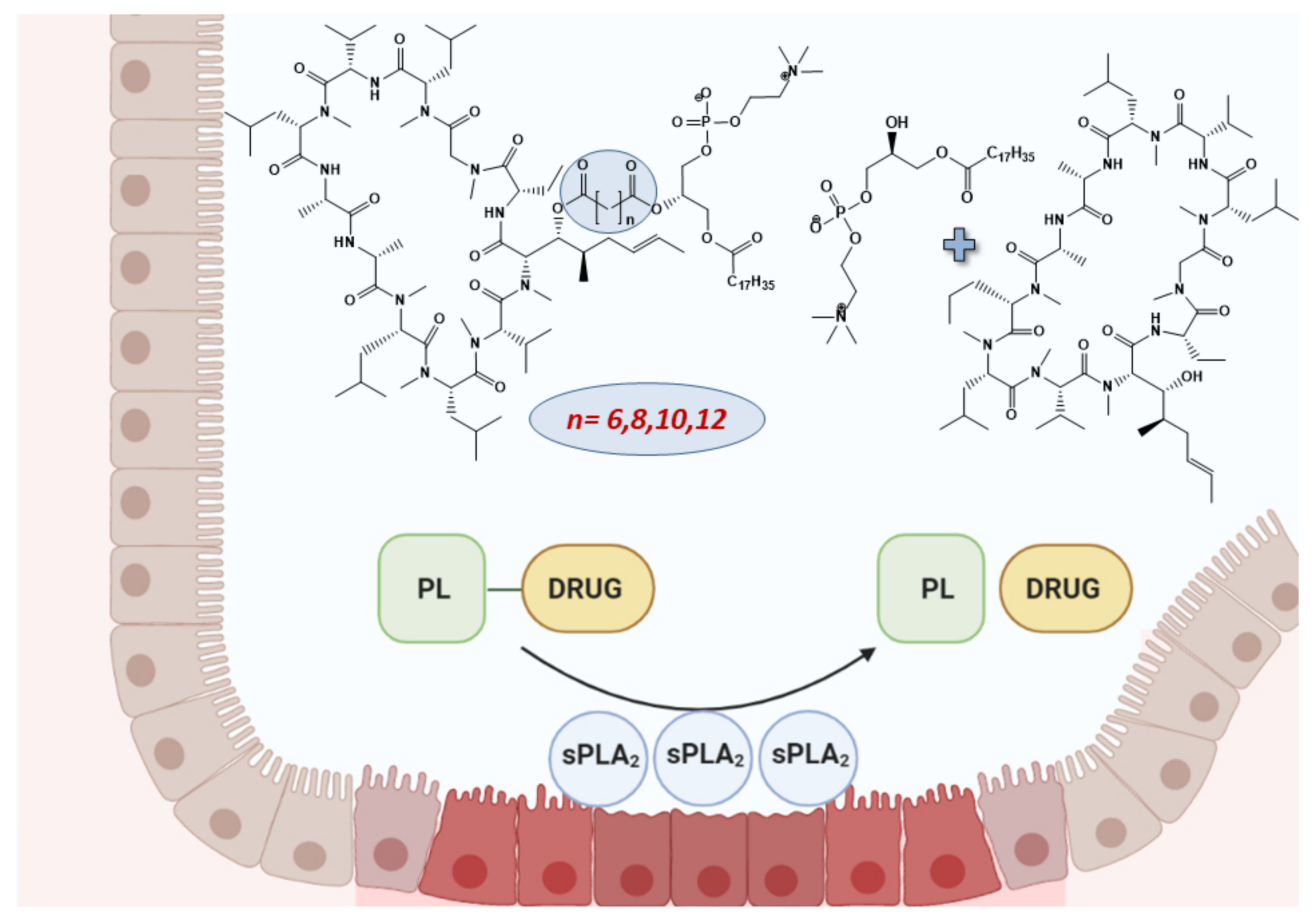
Illustration of PLA_2_-mediated prodrug activation in the inflamed intestinal epithelium, and chemical structures of the prodrugs with their activation products PL and cyclosporine. PLA_2_, phospholipase A_2_; PL, phospholipid.

Phospholipase A_2_ (PLA_2_) is an enzyme with significantly elevated expression/activity in the inflamed intestinal tissues of patients with IBD [19,20,21,22,23]. PLA_2_, in contrast to other phospholipases, specifically recognizes the *sn*-2 acyl bond of phospholipids (PL) and catalytically hydrolyzes this bond, thus liberating a fatty-acid (FA) and a lysophospholipid (LPL). Our drug targeting approach includes prodrug design in which the *sn*-2 positioned FA of the PL is replaced with the drug cyclosporine [18]. In this manner, PLA_2_ is to be used as the prodrug-activating enzyme, releasing the free drug from the PL-complex. Owing to the elevated levels of the enzyme, orally delivered PL-cyclosporine prodrugs will release the free drug specifically at the inflamed sites, effectively targeting the regions of intestinal inflammation, as illustrated in Figure 1.

The chemical connection between the cyclosporine and the PL influences the level of recognition and activation by PLA_2_ and hence the prodrug success, as we have shown previously in in vitro proof-of-concept studies [24,25]. In our previous study we reported a design, synthesis, and characterization for four novel PL-cyclosporine prodrugs with increasing linker length between the PL and the drug moiety (6, 8, 10 and 12 -CH_2_ units; Scheme 1) [18].

For the purpose of evaluating prodrug activation and potential to release the free drug in the areas with high PLA_2_ levels, a novel enzymatically enriched model was developed, the colonic brush border membrane vesicles (cBBMVs). In this model, tissue vesicles were produced from the colon’s mucosa of rats with induced colitis (vs. healthy animals). This unique model allowed us to study the different prodrug activation and to depict the optimal PL–linker–cyclosporine combination which will allow the greatest activation by PLA_2_ in a setting containing different enzyme levels reflective of the inflamed vs. healthy tissues in the colon.

## 2. Materials and Methods

### 2.1. Materials

Dextran sulfate sodium was purchased from TDB Consultancy AB, Uppsala, Sweden. Anti-sPLA_2_ antibody, anti-GAPDH antibody, secondary donkey anti-rabbit IgG antibody and immunoblotting reagents/Hybond ECL^TM^ nitrocellulose membrane were purchased from Abcam (Cambridge, MA, USA), Santa Cruz Biotechnology (Dallas, TX, USA), Santa Cruz Biotechnology (Dallas, TX, USA), and Biorad (Rishon LeTsyon, Israel), respectively. A series of four phospholipid–cyclosporine prodrugs were synthesized using two steps by condensation of the phospholipid to the cyclosporine through di-acyl chloride linkers with diverse lengths (6, 8, 10 and 12 methylene units); all steps required for structure elucidation and purity were completed, as demonstrated in our previous work [18]. Isopropanol, methanol, and water (Merck KGaA, Darmstadt, Germany) were of ultra-performance liquid chromatography (UPLC) grade. All other chemicals were of analytical reagent grade.

### 2.2. Dextran Sulfate Sodium Animal Model

The dextran sulfate sodium (DSS) model of acute colitis in male Wistar rats was optimized using previously published reports, with minor modifications [26,27]. Animal studies were conducted according to the protocols approved by the Animal Use and Care Committee of the Ben-Gurion University of the Negev (Protocol IL-36-05-2019(C)). The animals (male Wistar rats weighing 230–260 g, Harlan, Israel) were housed and handled in agreement with the Ben-Gurion University of the Negev’s Unit for Laboratory Animal Medicine Guidelines.

Briefly, the 5% DSS (molecular weight, 40,000 g/mol) was introduced in the drinking water of the rats to form 5% *w/v* solution for 9 days. The control animals drank water only. The clinical progression of colitis was evaluated daily using the disease activity index (DAI) score, which includes the following parameters: weight loss compared with initial weight, stool consistency, and rectal bleeding. DAI parameters are defined as: weight loss 0 (no loss), 1 (1–5%), 2 (5–10%), 3 (10–20%), and 4 (>20%); stool consistency: 0 (normal), 2 (loose stool), and 4 (diarrhea); and bleeding: 0 (no blood), 1 (hemoccult positive), 2 (hemoccult positive and visual pellet bleeding), and 4 (gross bleeding, blood around anus). Prior to the experiment the rats were fasted overnight (12–18 h) with free access to water. On day 9, the rats were anesthetized by intramuscular injection of 1 mL/kg of ketamine-xylazine solution (9%:1%). The abdomen was opened by a midline incision of 3–4 cm, the length of the colon was measured, followed by the colon removal. Upon sacrifice and colon examination, macroscopic damage score and disease severity scores were blindly assessed based on rectal bleeding, rectal prolapse, stool consistency and blood.

### 2.3. Colonic Brush Border Membrane Vesicles (cBBMVs) Production

Colonic brush border membrane vesicles (cBBMV) were prepared by Ca^2+^ precipitation from the colonic mucosal tissues of male Wistar rats according to previously published protocols for small intestinal vesicles with some modifications [28,29,30,31]. Briefly: (1) Large intestines were washed with ice cold saline and separated from mucus; (2) The intestinal mucosa was scraped from the luminal surface onto the ice-cold glass slides and placed into buffer containing 50 mM KCl and 10 mM Tris-HCl (pH 7.5, 4 °C); (3) The samples were then homogenized (Polytron), 10 mM CaCl_2_ was added, and the homogenate was placed on a shaker for 30 min at 4 °C; (4) Following Ca^2+^ precipitation, the samples were split into 4 tubes and purified by centrifugation at 10,000× *g* for 10 min, following the separation of the supernatant and two additional purification steps: centrifugation at 48,000× *g* for 30 min and re-suspension of the pellet in 300 mM mannitol, 10 mM HEPES/Tris (pH 7.5) and centrifugation at 24,000× *g* for 60 min. The quality of the cBBMV purification was tested using the brush border membrane enzyme markers gamma-glutamyl transpeptidase (GGT) and alkaline phosphatase (ALP). The cBBMVs were used fresh. In future replication of this experiment, repeated thawing should be avoided, and enzyme levels should be evaluated prior to each use.

### 2.4. PL-Cyclosporine Prodrug Activation in cBBMV

Stock solutions of PL–cyclosporine conjugates were prepared in methanol. Purified cBBMVs were incubated with the prodrug solutions in MES buffer (50 mM pH 7.4) at 37 °C for 90 min. Triplicate samples (each sample 50 µL) were taken at time 0, 15, 30, 45, 60, 90, 120 and 180 min. The reaction was stopped with addition of 0.1 mL acetonitrile, and the samples were centrifuged at 4000× *g* for 10 min, and immediately assayed with HPLC/UV. Both vesicles derived from diseased and healthy rats were produced from colons of 10 rats each. Values are expressed as means ± standard deviation (SD).

### 2.5. PLA_2_ Quantification by Immunoblotting

PLA_2_ presence was determined in both cBBMVs and mucosal homogenate in diseased vs. healthy rats. The loaded amount of protein was 25 µg, and both homogenate and consequently colonic brush border membrane vesicles (CBBMV) were obtained from colon mucosa of 10 rats for both diseased and healthy rats [32]. The protein content was determined using the Bradford assay. Samples were resolved in 15% sodium dodecylsulphates gel electrophoresis, followed by electrophoretic transfer onto a Hybond ECL nitrocellulose membrane. The membrane was blocked for 1 h in TTBS solution with 3% milk, followed by overnight incubation at 4 °C with rabbit polyclonal anti-PLA_2_ antibody (1:1000). The secondary antibody was donkey anti-rabbit IgG (1:5000). Equal protein loading was ensured by normalization to GAPDH (glyceraldehyde 3-phosphate dehydrogenase). Detection of immunoreactive bands was carried out using enhanced chemiluminescence. The blots were subsequently visualized with ECL reagent and developed using an Agfa Curix 60 developing machine. Semi-quantitative analysis was carried out using a computerized image analysis system (Image Studio Lite Ver. 5.2).

### 2.6. Analytical Methods

Visualization of cBBMV was performed on Transmission Electron Microscope Tecnai T12 G^2^ TWIN at liquid nitrogen temperatures. High-performance liquid chromatography (HPLC) analyses were performed on a Waters (Milford, MA, USA) Acquity HPLC system equipped with photodiode array detector and Empower software. We developed a method for prodrug determination using a Waters XBridge C8 3.5-μm 4.6 × 150 mm column, mobile phase consisting of 70:3:27 (*v*/*v*) isopropanol:methanol:water over 10 min (flow rate, 0.5 mL/min), at detection wavelength 210 nm and retention times 6.2, 6.4, 7.0 and 7.3 min for PL-cyclosporine conjugate containing 6-, 8-, 10- and 12-carbon linker, respectively. Injection volumes for all HPLC analyses were 10 μL. Standard curves were carried out in buffer–methanol (1:1) solution prior to the experiment (R^2^ > 0.999). The minimum quantifiable concentration for all prodrugs was 10 ng/mL. The inter- and intra-day coefficients of variation were <1.0 and 0.5%, respectively.

### 2.7. Statistical Analysis

The difference in the macroscopic score and colon length between colitis-induced and healthy animals were determined as statistically significant through Student’s *t*-test n = 10/group. Results for both DSS and healthy animals are presented as mean ± SD and *p* < 0.05 was termed significant. Enzyme enrichment in the cBBMVs from healthy and diseased animals are presented as mean ± SD, (n = 4). Results for PL–cyclosporine activation in cBBMVs for both DSS-treated and healthy animals are presented as mean ± SD; n = 4, per each phospholipid-cyclosporine conjugate. All statistical analyses were performed using Graph-Pad Prism 9.2.0 software (La Jolla, CA, USA).

## 3. Results

### 3.1. Dextran Sulfate Sodium Animal Model

The evaluation of disease progression in the DSS-induced model of colitis in rats was demonstrated through DAI, macroscopic disease severity score and shorter colon lengths in the diseased group, compared with healthy rats, as per previous reports [26,27].

**Figure 2:**
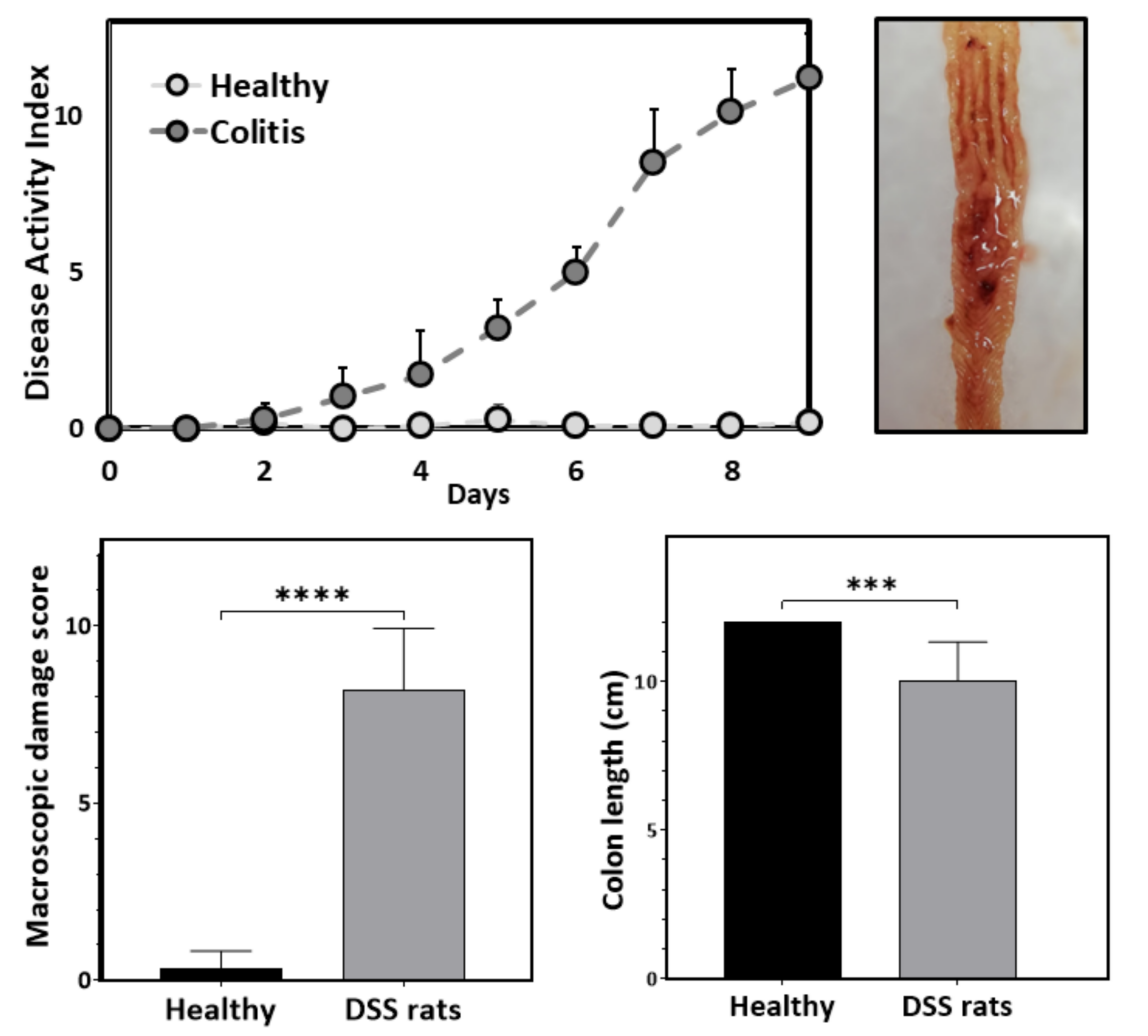
Disease activity index (upper left panel) in DSS-induced colitis rats; macroscopic presentation of the colonic luminal surface of DSS-rats (upper right panel); macroscopic damage score (lower left panel) and colon length (lower right panel) in DSS-induced colitis rats vs. healthy animals. Data are presented as mean ± SD, n = 10, *** *p* ≤ 0.001, **** *p* ≤ 0.001. DSS, dextran sulfate sodium.

Within 9 days, rats gradually developed signs of acute colitis; in most animals, weight loss was >20%, and diarrhea, and gross bleeding from the anus occurred. The measured disease activity index (DAI) on day 9 resulted in the score of 12 for diseased animals, vs. no symptoms and a DAI score of ~0 for healthy animals, used as a control (Figure 2, upper, left panel). On day 9, the rats were sacrificed, and colons were harvested. Macroscopic presentation of the colonic luminal surface is presented in Figure 2, upper right panel. There was a significant difference in the macroscopic disease severity scores (rectal bleeding, rectal prolapse, diarrhea, colonic bleeding) between colitis-induced (*M* = 8.22, SD = 1.716) and healthy animals (*M* = 0.33, SD = 0.5); t (9.35) = 13.24, *p* < 0.0001 (Figure 2, lower left panel). We observed shorter colon length in the diseased, colitis group (*M* = 10.1, SD = 1.28) compared with healthy animals (*M* = 12.45, SD = 0.83); t (18) = 4.97, *p* < 0.0001, (Figure 2, lower, right panel), which was in agreement with previous reports [26,33].

### 3.2. Colonic Brush Border Membrane Vesicles (cBBMV)

The cBBMVs, produced from both diseased and healthy colons, were enzymatically tested, as presented in Table 1.

**Figure 3:**
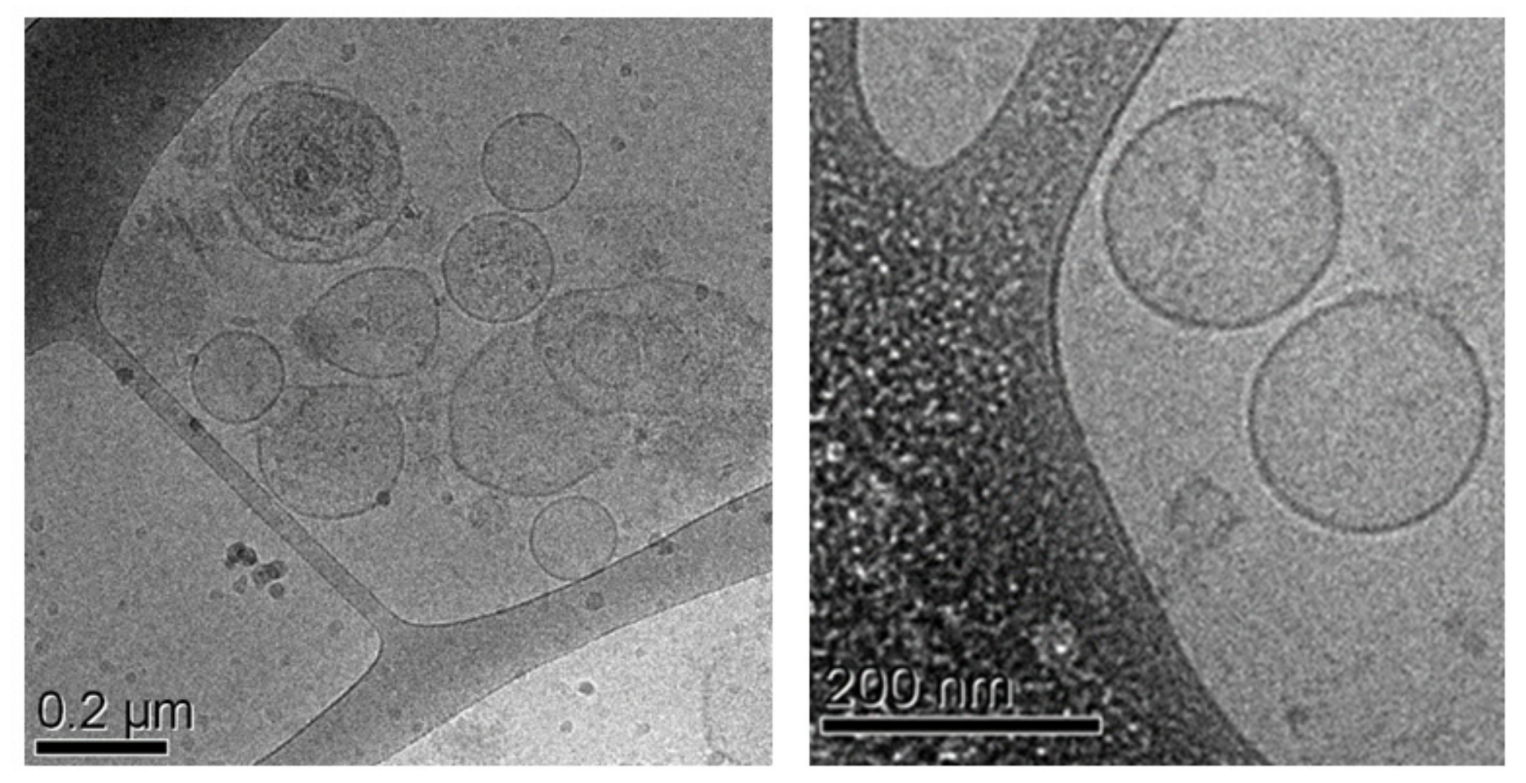
CryoTEM visualization of cBBMVs. CryoTEM, cryogenic transmission electron microscopy; cBBMV, colonic brush border membrane vesicles.

The quality of the cBBMVs was confirmed by measuring the enrichment of the enzymes alkaline phosphatase (ALP) and gamma-glutamyltransferase (GGT), with 4–5-fold higher levels of ALP and GGT in the cBBMVs vs. the colonic mucosal tissue homogenate, in both diseased and healthy colons. Freshly produced vesicles were visualized under transmission electron cryomicroscopy (CryoTEM), Figure 3; uniform size and shape were demonstrated in both vesicles from diseased and healthy animals. The vesicle size from healthy colons was 210.4 ± 90.2 nm (n = 50), and vesicles derived from diseased animals were 212.8 ± 91.3 nm (n = 50).

### 3.3. Immunoblotting

**Figure 4:**
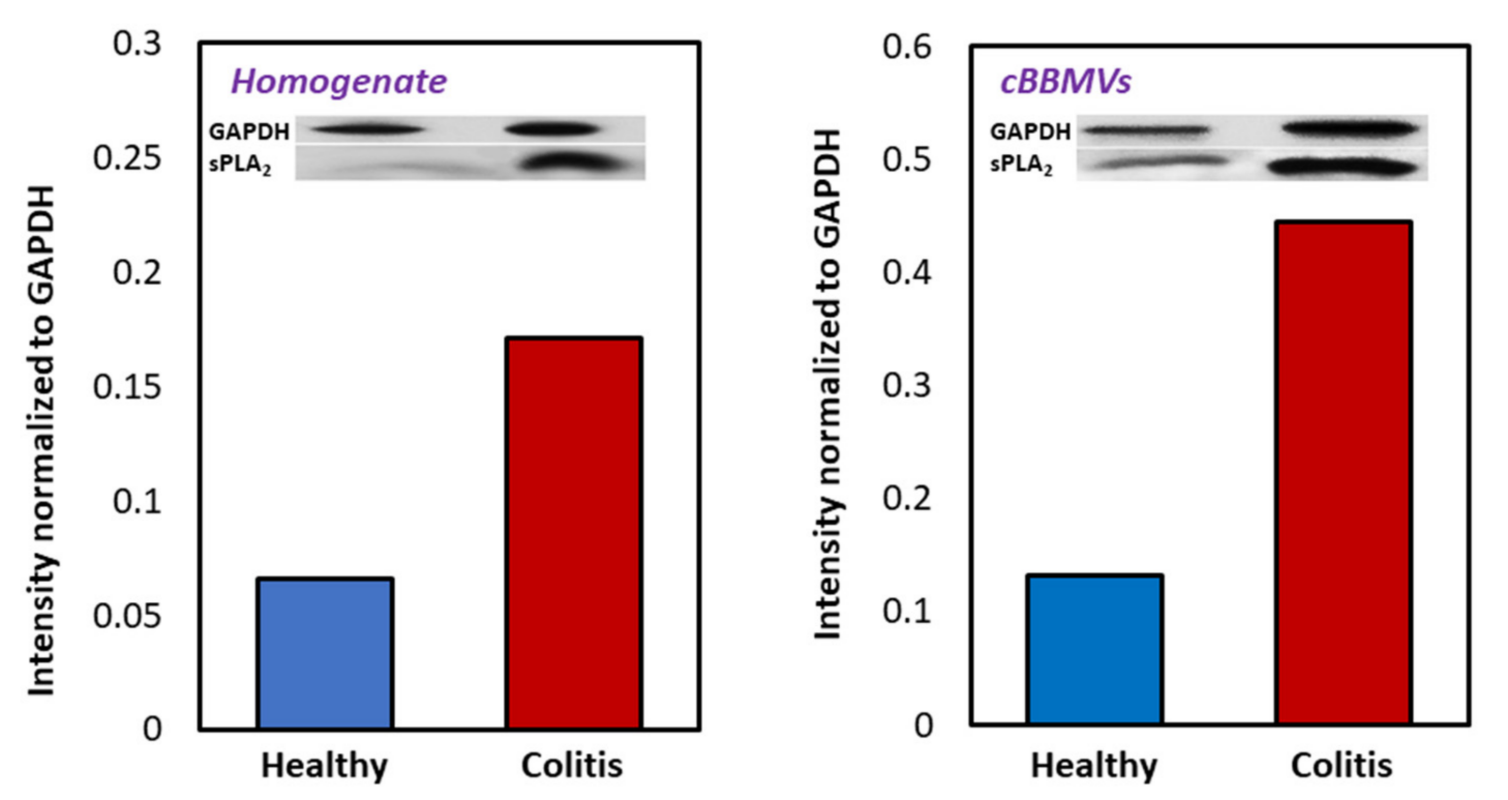
Immunoblotting results for PLA_2_ levels in colonic mucosal homogenate (**left**) and cBBMVs (**right**), obtained from a pool of animals in diseased vs. healthy rats (n = 10) normalized to GAPDH. PLA_2_, phospholipase A_2_; cBBMV, colonic brush border membrane vesicles; GAPDH, glyceraldehyde 3-phosphate dehydrogenase.

Immunoblotting of cBBMVs showed 3.4-fold higher expression of PLA_2_ enzyme in the diseased vs. healthy vesicles (Figure 4, right panel). The intensity of bands demonstrated 2.6-fold higher expression of PLA_2_ in the mucosal homogenate of diseased vs. healthy rat colons, which was the staring material for cBBMV production (Figure 4, left panel).

### 3.4. Prodrug Activation Study in cBBMVs

**Figure 5:**
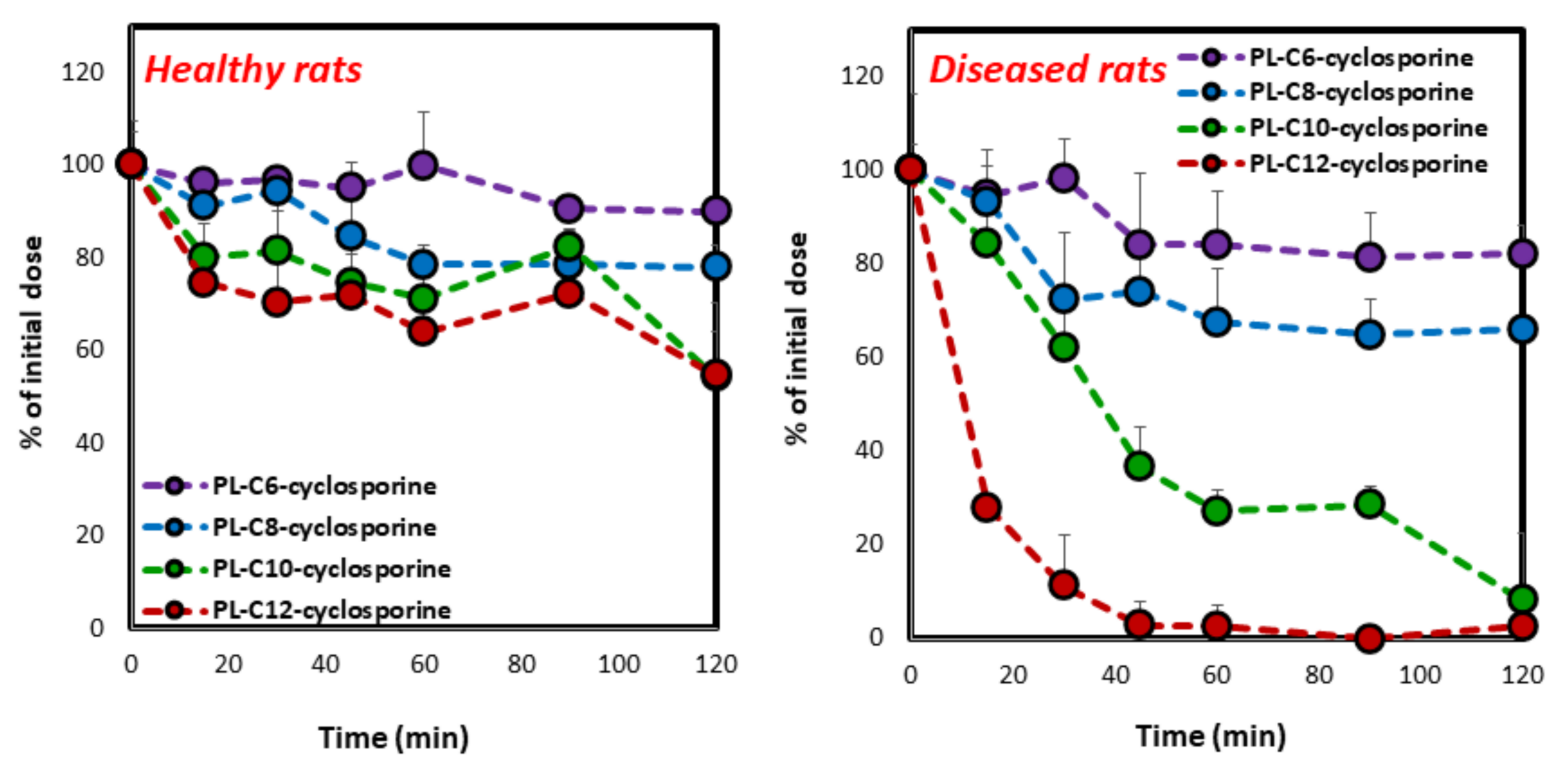
Activation of the four PL–cyclosporine prodrugs following incubation with healthy (**left**) vs. diseased (**right)** cBBMVs. Average ± SD; n = 4. PL, phospholipid; cBBMV, colonic brush border membrane vesicles.

Activation of the four PL-cyclosporine prodrugs (i.e., liberation of free drug) differing by their linker length (6, 8, 10, and 12 -CH_2_ units) throughout 120 min incubation in the freshly produced cBBMVs from healthy (left panel) vs. diseased colons (right panel) is demonstrated in Figure 5. The significant differences between prodrug activation by diseased vs. healthy colonic vesicles can be readily appreciated; while cBBMVs from healthy colons induced only marginal activation, substantial prodrug activation was evident by colitis-derived cBBMVs. Together with the PLA_2_ overexpression, these data validate our drug targeting strategy.

The rate and extent of activation of the four prodrugs varied significantly by linker length, especially in the diseased cBBMVs. While quick and complete activation of the entire dose was obtained for the 12-carbon linker prodrug, slow and much less overall activation was obtained for the 6/8-carbon linkers.

## 4. Discussion

The traditional prodrug approach focuses on altering various physicochemical features of the parent drug through binding to hydrophilic/lipophilic functional groups in order to enhance drugs’ solubility or passive permeability [34,35,36,37]. More recently, modern prodrug strategies, where promoieties are attached to the parent drug aiming to target specific membrane transporters/enzymes, are increasingly employed [38,39]. This approach considers molecular/cellular parameters such as membrane transporter influx/efflux or enzyme expression and distribution, creating an opportunity for site specific drug targeting [40,41]. In this work we applied this modern biopharmaceutical approach to allow drug targeting specifically to the inflamed sites throughout the GIT of IBD patients. This offers the major clinical advantage of efficient treatment of any localization throughout the GIT. Additionally, this approach will allow the extension of the therapeutic index of clinically significant drugs, such as cyclosporine, making our approach to IBD targeting unique and valuable in the search for improved drug therapy and overall patient care.

The system for our prodrug activation studies was developed based on the brush border membrane vesicles (BBMVs) [28,30,31]. The rationale for the use of brush border membranes lies in the fact that their use could provide results that approximate metabolic and morphologic functionality of the intestinal brush border [42]. In this study, we applied two major changes to this method. Primarily, based on extensive literature search, this is the first time BBMVs were produced from animals in diseased state, which makes it possible to study diseased vs. healthy intestine, allowing the revelation of important disease-specific characteristics, both in the context presented in this work (i.e., prodrug activation) and in many other settings. cBBMVs are an enzymatically enriched model, which in this case was able to provide sufficient overexpression of secretory PLA_2_, to reflect the inflamed intestinal tissues. Moreover, while BBMVs are traditionally produced from the small intestine, it is far less common to apply this model to the colon; the murine model of gastrointestinal inflammation (DSS-induced colitis) is characterized by acute inflammation mainly present in the colon, which is why the mucosal tissues were harvested from this organ, instead of the usual small intestinal mucosa [29,30,31]. The enzyme enrichment, size and shape of cBBMVs produced from colons of DSS-induced colitis, in addition to healthy rats, established the quality of this experimental method. The overexpression of our therapeutic target, PLA_2_ enzyme (Figure 4), confirmed the feasibility of our strategy for drug targeting in IBD, and made it a suitable model for testing the PL-based prodrugs.

Activation of PL-cyclosporine prodrugs containing variable linker length within the cBBMVs produced from diseased colons highlighted the gradual increase in the activation rate by increasing the length of the carbonic linker between the PL backbone and the drug (Figure 5, right panel). On the other hand, incubation with cBBMV from healthy rat colons demonstrated significantly less activation, although some activation was present due to the basal physiological levels of PLA_2_ in the intestine (Figure 5, left panel). The mechanism of this linker length effect on prodrug activation pattern lies in the steric hindrance; the PLA_2_-mediated activation of the prodrug is highly reliant on the prodrug structure (spatial arrangement of the drug) and fitting into the transition state geometry of PLA_2_, which dictates the binding between the prodrug and the enzyme. In fact, it was previously proposed that PLA_2_ only hydrolyses PL when the *sn*-2 position is occupied by fatty acid [43]. We have shown, however, that this is true when the drug is linked directly to the sn-2 position [44], but with the proper spacer between the PL and the drug moiety, the enzymatic activation can occur after all [24,25,45]. It is interesting to note that for PL-based prodrugs of smaller drugs (diclofenac and indomethacin), 6-carbon linker was found to be optimal, while both shorter or longer linkers hampered the prodrug activation [24,25]; this shows that the optimal molecular design of PL-based prodrugs is changing and depends on the size, volume and the 3D assembly of the specific parent drug in question.

Prodrugs utilizing lipids (such as PL) as carriers showed the ability to join the physiological lipid trafficking pathways [46], target the specific step in lipid processing (especially if this pathway is altered in disease) and facilitate drug release at a specific target site [40,47]. The increased levels of PLA_2_ in the diseased tissue will lead to increased amounts of free drug in the actual inflamed intestinal patches, accompanied by decreased drug levels in non-diseased tissues, resulting in an extended therapeutic index and improved drug therapy with this important immunosuppressant. Importantly, a great clinical advantage is offered by our drug targeting approach: the inflammation localization varies in IBD patients, and since up-to-date IBD drug products target a general intestinal region (mainly the colon/lower ileum), these products will not be effective if the inflammation is outside the targeted region (e.g., upper small intestine in Crohn’s disease patients). Since our approach exploits a feature innate to the inflamed tissue(s) per se (i.e., PLA_2_ overexpression), following a suitable prodrug formulation that may circumvent the stomach environment, efficient treatment of inflamed areas throughout the GIT may be allowed. Offering a potential solution to this unmet need, together with extending the therapeutic index of clinically significant drugs, may identify the advantageous potential of our approach for drug targeting in IBD.

Additionally, some premature activation may occur in the proximal intestinal segments due to activation by pancreatic PLA_2_ [48]. This enzyme is present in low, basal physiological levels in the healthy small intestine [49] and depending on the prodrug structure it is insufficient to significantly activate PL–cyclosporine prodrugs, while overexpression in the disease state may allow quick and complete activation. Indeed, our previous work with PL–diclofenac prodrugs further demonstrate that PL-based prodrugs are stable even after 5 h in healthy rat small intestinal fluid [25]. These points indicate that a good targeting capacity can be accomplished throughout the gut.

Limitation of our approach lies in the fact that individual optimization in terms of the prodrug structure and linker length is needed for each parent drug, as demonstrated in our previous work [18,24,25,45]. Indeed, higher molecular weight drugs such as cyclosporine might require longer linkers for optimal activation [50] than the ones with lower molecular weight [45,51,52,53]. Nevertheless, advanced in-silico techniques are nowadays available, and we and others have previously shown just how useful they may be in optimization of the prodrug structure [45,50,51,52,54,55,56]. Future avenues should explore the pharmacological effects of PL–cyclosporine prodrugs in novel genetically engineered or humanized IBD animal models, in addition to development of suitable formulations that would circumvent the gastric environment.

Lastly, since the overexpression of PLA_2_ occurs in many malignant and inflammatory conditions (i.e., rheumatoid arthritis, colorectal cancer, vascular inflammation), this prodrug approach has the potential to improve the treatment of various other diseases [57,58,59].

## 5. Conclusions

Our PL-based prodrug approach for IBD drug targeting was shown to deliver the free drug selectively to inflamed intestinal regions. This approach holds significant clinical advantage of efficient drug targeting of any localization throughout the GIT. Additionally, extended therapeutic index of clinically significant drugs may be achieved, maximizing cyclosporine levels in the inflamed intestinal tissues while minimizing systemic immunosuppression, thereby making our IBD targeting approach valuable in the search for improved drug therapy and overall patient care.

## Figures and Tables

**Table 1:** Enzyme activity and enrichment factors in the cBBMV from healthy rat colon and DSS-induced colitis rats. Data are presented as mean ± SD, (n = 4). cBBMV, colonic brush border membrane vesicles; DSS, dextran sulfate sodium.

**Healthy Rats**			
	Specific activity (U/mg protein)		Enrichment factor
**Enzyme**	**Homogenate**	**cBBMV**	
**Alkaline phosphatase**	8.08 ± 0.39	36.97 ± 0.26	4.58 ± 0.25 (n = 4)
**Gamma-glutamyltransferase**	6.37 ± 1.07	25.35 ± 0.13	4.03 ± 0.65 (n = 4)
**DSS-induced colitis rats**			
	Specific activity (U/mg protein)		Enrichment factor
**Enzyme**	**Homogenate**	**cBBMV**	
**Alkaline phosphatase**	12.35 ± 0.12	38.56 ± 0.63	3.12 ± 0.08 (n = 4)
**Gamma-glutamyltransferase**	1.16 ± 0.23	6.29 ± 0.12	5.54 ± 1.23 (n = 4)
